# The Most Popular Videos Promoting Breast Enhancement Products on TikTok: Cross-Sectional Content and User Engagement Analysis

**DOI:** 10.2196/73336

**Published:** 2025-06-06

**Authors:** Jing Lin, Wanlin Li, Lian Zhu, Ning Li, Shi Chang

**Affiliations:** 1 Department of General Surgery, XiangYa Hospital, Central South University, No.87 XiangYa Road, Hunan, Changsha, 410008, China, 86 18119122648, 86 073189753999; 2 Department of General Surgery, The 3rd Affiliated Teaching Hospital, Xinjiang Medical University, Urumqi, China; 3 Furong Laboratory, Changsha, China; 4 National Engineering Research Center of Personalized Diagnostic and Therapeutic Technology, Changsha, China

**Keywords:** body image, breast enhancement, TikTok, social media, content analysis, engagement analysis, misinformation

## Abstract

**Background:**

The proliferation of health-related content on social media platforms has changed the way people access and interpret information about cosmetic medicine. TikTok (ByteDance) has become an important platform for sharing breast enhancement content, yet little is known about the quality, credibility, and impact of such information on user perceptions and decision-making.

**Objective:**

This paper aims to analyze the characteristics of breast enhancement videos, including uploader demographics, product details, promotional claims, and user engagements, to better understand the nature of the claims and products encountered by users.

**Methods:**

We conducted a cross-sectional content analysis of the top 150 most-liked breast enhancement videos via TikTok’s web interface. The videos were coded according to the uploader’s traits (gender expression and account type), product details (type and scientific evidence), and promotional strategies (testimonials and sponsorship disclosures). Engagement metrics (likes and shares) were recorded, and nonparametric tests (Mann–Whitney *U* test) were used to compare the engagement between licensed physicians and uncertified content creator uploaders. Descriptive statistics were calculated for all the variables.

**Results:**

Overall, 85 videos were included in the final analysis, with most uploaders presenting a feminine gender expression (59/85, 69.4%) and using uncertified content creator accounts (59/85, 69.4%). The most promoted product types were breast enhancement creams or oils (32/85, 37.6%) and breast implants (22/85, 25.9%). Most videos (71/85, 83.5%) depicted the products positively; however, most videos (78/85, 91.8%) provided no scientific evidence of the product’s efficacy. Engagement metrics revealed that videos by licensed physicians received significantly higher thumbs up (median 9761, IQR 4975-19,492) than uncertified content creator uploaders (median 701, IQR 280‐2604; *P*=.002). Only one video (1.2%) of the 85 videos included a “before and after” visual component, and most videos (75/85, 88.2%) omitted product purchasing details. Sponsorship disclosures were absent in most of posts (79/85, 92.9%).

**Conclusions:**

TikTok’s short video format fosters widespread and rapid dissemination of breast enhancement information, representing a key strength in democratizing health communication. Its user-friendly interface and visual appeal also offer a valuable avenue for medical professionals to engage audiences more dynamically. However, the lack of rigorous content checks can amplify misleading or unverified claims. To address these weaknesses, implementing dual-mode content review could be essential for maximizing TikTok’s capacity to support informed public health decision-making.

## Introduction

### Breast Enhancement

Breast size and shape have significant psychological importance for many women, affecting body image, self-perception, and confidence [1,2]. Research consistently shows that breast appearance concerns are widespread globally, with the Breast Size Satisfaction Survey documenting that approximately 70% of women across 40 countries experience discrepancies between their ideal and actual breast size [1].

These concerns can be particularly pronounced among specific populations, including those who have undergone mastectomy for breast cancer treatment [3].

Breast enhancement encompasses various procedures and products aimed at increasing breast size or improving shape. Surgical breast augmentation has become the second most common cosmetic surgical procedure worldwide and the most common among women [4]. In China, studies indicate a growing interest in breast enhancement procedures, with women aged 19‐53 years (mean 31.2, SD 6.8 y) seeking these interventions [3]. This trend exists within a broader social context in which various media outlets emphasize women’s physical appearance, particularly breast aesthetics, as central to feminine attractiveness [5,6].

### TikTok’s Influence on Body Image

In recent years, social media platforms have reshaped the way beauty norms are established and disseminated. TikTok (ByteDance), with its short-form videos and algorithmic recommendation system, offers a highly personalized user experience; as a result, content focusing on aesthetic ideals can rapidly circulate to large audiences [7,8]. The platform’s recommendation system creates a personalized user experience that can amplify beauty-focused content, potentially intensifying exposure to specific beauty ideals [8]. Under the breast enhancement hashtag, numerous videos promote procedures, supplements, creams, and other methods purportedly leading to fuller or more shapely breasts [9]. These products and techniques are often marketed as accessible solutions for attaining desired breast aesthetics. However, many of these promotional materials lack robust scientific validation, raising concerns about consumer safety, potential health risks, and ethical implications [10-12]. This digital landscape highlights social media’s considerable influence on beauty standards and their psychological impact, particularly among younger users.

### Theoretical Framework: Social Comparison and Cultivation Theory

Media psychology research provides insights into why TikTok could strongly affect viewers’ body perceptions. “Social Comparison Theory,” originally proposed by Festinger [13], posits that individuals assess their worth by comparing themselves to others. On a platform overflowing with curated visuals, users frequently measure their bodies against influencers displaying “ideal” breast sizes and shapes, potentially intensifying body dissatisfaction and perceptions of inadequacy [11,14].

Beyond social comparison, “Cultivation Theory”[15] argues that repeated media exposure shapes individuals’ perceptions of reality. Continuous engagement with abundant breast enhancement content may overstate the prevalence and desirability of such interventions, generating an environment where specific breast shapes become the perceived norm. This repetition fosters unrealistic expectations and internal pressure to conform to media-driven standards of ideal breast appearance.

### Research Gaps

Previous research has extensively explored social media’s influence on body image, particularly in areas such as skin lightening and muscle building; however, there is a notable lack of studies investigating breast enhancement content on platforms, including TikTok. Studies on skin-lightening products, for instance, reveal inadequate disclosure of associated risks, pointing to the potential dangers of misleading internet-based health information [12]. Similarly, research on TikTok’s promotion of breast enhancement products remains scarce. This gap in the literature is particularly significant in the Chinese context, where representative surveys of women’s preferences related to breast augmentation and influencing factors in decision-making remain limited compared to Western populations [16-22].

### Study Objectives

We investigated the characteristics of breast enhancement content on TikTok through a content analysis of the most popular posts under the breast enhancement hashtag . We sought to provide a comprehensive understanding of the portrayal of breast enhancement on TikTok by analyzing the demographic profiles of the content uploader, the content of breast enhancement, and efficacy claims. Furthermore, we evaluated user engagement metrics to explore how these videos influenced the audience’s perceptions and their contribution to the dissemination of beauty ideals. A content analysis methodological approach is warranted due to TikTok’s extensive reach and its potential to exacerbate body dissatisfaction among users. Previous studies have demonstrated that this method enables researchers to categorize and interpret media representations systematically [23,24]. Ultimately, we aimed to offer critical insights into the interplay between social media, body image, and user engagement within the context of breast enhancement through our findings in this study.

## Methods

### Ethical Considerations

This research did not involve the use of clinical data, human participants, or laboratory animals. All data were obtained from publicly accessible TikTok videos, and fully anonymized; no user IDs, usernames, or identifiable personal information were recorded or included in the manuscript or supplementary materials. As there was no interaction with users, an ethics review was deemed unnecessary.

### Coding Procedure

A total of 2 researchers (WL and JL) identified and compiled a list of hashtags related to breast enhancement. We initially analyzed the number of videos per hashtag on January 1, 2025, with initial searches for breast augmentation (105 videos), breast lift (142 videos), breast improvement (139 videos), and breast enhancement (197 videos). Due to the large number as well as overlap of videos, we selected the hashtag breast enhancement, which had the highest number of videos for our analysis because it focused on the claim to increase breast size. This keyword is consistent with previous studies [25-27]. In the study, the researchers focused on the top 150 most-liked videos on the Chinese version of TikTok, which collectively received 1,802,462 thumbs up during data collection.

### Data Collection

Data were gathered using TikTok’s official web version. A new account was created to eliminate potential biases owing to the browsing history on the search results. Following established methodologies [14,20,22], the 150 most popular videos were compiled into a secure spreadsheet accessible only to the research team. Our codebook labels were developed through a systematic process designed to capture the dimensions of breast enhancement content on TikTok. The development began with a research assistant and principal investigator independently reviewing the first 30 videos using inductive analysis to identify emerging content patterns. Label selection was guided by 3 considerations: alignment with existing social media content analysis frameworks, relevance to our research questions examining product characteristics and promotional strategies, and conceptual distinctiveness between categories. We included labels such as “product type,” “efficacy claims,” and “risk disclosure,” based on their significance in analyzing potentially misleading health content, excluding categories like “shapewear bras” after pilot coding showed these were conceptually distinct from methods purporting to create lasting changes.

Following refinement through pilot testing, 2 trained research assistants independently viewed 10 videos per week, each at least three times, by applying the coding scheme we identified. This approach ensured that our labels captured the multifaceted nature of breast enhancement content while maintaining analytical precision across the 85 videos that met our inclusion criteria. Mental health support was provided for coders throughout this process who found the content uncomfortable or had low self-esteem, acknowledging the potentially sensitive nature of the content.

### Data Extraction

Each video was analyzed to extract data on the uploader’s characteristics (such as gender expression and uploader authentication), product details (including product type and scientific evidence), and promotional elements (including testimonials and sponsorships).

### Coding Framework

The coding framework was adapted from previous studies on social media content analysis [12,28], with modifications tailored to the context of breast enhancement content on TikTok.

### Coding Reliability

During the coding period, the percentage agreement between coders was calculated weekly, with an average consistency rate of 80% across coding categories and weeks. There are no established thresholds for acceptable reliability in qualitative research; nevertheless, prior studies suggest that intercoder reliability exceeding 80% reflects strong consensus [29].

Inter-rater reliability was measured using percent agreement rather than kappa statistics because of the susceptibility of the study to the limitations of kappa coefficients, a phenomenon in which high agreement rates may yield low kappa values, particularly in datasets with skewed distributions or asymmetrical coding patterns [30-32]. Following Miles and Huberman’s [33] recommendation of 80% agreement as an acceptable standard, we maintained a rigorous weekly assessment of coding consistency across all content categories.

To maintain coding reliability, we implemented several measures: (1) weekly calibration meetings where the 2 coders and a third researcher convened to address coding disagreements and refine the final codes to ensure accuracy, (2) refinement of the coding framework after each reliability assessment, and (3) random double-coding of 25% of content each week to identify potential coding drift. For categories showing lower reliability scores in the early weeks, we provided coder training again with clarified operational definitions and exemplary cases.

There is considerable inconsistency in the interpretation of intercoder reliability results in qualitative research, as noted by O’Connor and Joffe [29]. Following Landis and Koch’s [34] interpretive framework, our initial week’s agreement (78%) would be classified as “substantial agreement,” while our final weeks consistently achieved “excellent agreement” [34]. By the final 2 weeks of coding, all content categories surpassed the 80% agreement threshold.

Descriptive statistical methods were employed to analyze the content of videos within the breast enhancement hashtag, focusing on the trends and patterns in the dataset.

### Coding Attributes

The data were organized into 3 primary categories using the coding framework as follows: uploader characteristics, product details, and promotional elements.

#### Uploader Characteristics

In terms of gender representation, they were classified as feminine, masculine, or unidentifiable. Verification status was categorized as licensed physicians, lawyers, self-media creators, for-profit companies, or uncertified content creators. Content format was assigned to 1 of 6 styles: monologue with and without speaker visibility, question-and-answer (Q&A) format, PowerPoint (PPT) or educational course, animated content, or television programs or documentaries.

Verification status was categorized as licensed physicians, lawyers, self-media creators, for-profit companies, or uncertified content creators.

Content format was assigned to 1 of 6 styles: monologue with and without speaker visibility, question-and-answer (Q&A) format, PowerPoint (PPT) or educational course, animated content, or television programs or documentaries.

#### Product Details

In terms of product classification, products were recognized as breast implants, enhancement creams, essences or oils, patches, capsules, protein drinks, powders or Chinese medicinal drinks, Chinese acupressure, enhancement instrument, needles, or homemade products. User satisfaction perception was rated as positive, neutral, or negative based on the tone of the video. Scientific validation claims were documented as assertions of scientific backing, claims of no evidence, or absence of such references. Visual comparisons were noted as present (including “before and after” visual) or absent. Purchase methods were tagged as offline brand purchase, direct internet-based purchase, profile linked website reference, or unspecified. Brand acknowledgment was recorded as explicit mention or omission.

User satisfaction perception was rated as positive, neutral, or negative based on the tone of the video. Scientific validation claims were documented as assertions of scientific backing, claims of no evidence, or absence of such references. Visual comparisons were noted as present (including “before and after” visual) or absent. Purchase methods were tagged as offline brand purchase, direct internet-based purchase, profile linked website reference, or unspecified. Brand acknowledgment was recorded as explicit mention or omission.

#### Promotional Elements

User testimonials were separated into claiming personal use of the product and those who do not. Endorsement disclosures were labeled as confirmed, without sponsorship, ambiguous (brand name visible), or unidentified. Content tone was classified as promotional (product marketing), informational (factual content), or confessional or oppositional (personal critique or negative experience).

### Statistical Analysis

Categorical variables were summarized as frequencies and percentages. Descriptive statistics, including medians and IQRs, characterized video metrics such as engagement and content attributes. Nonparametric analyses, specifically the Mann–Whitney *U* test, were applied to compare differences in engagement metrics (such as likes) between videos uploaded by licensed physicians and uncertified content creators, accounting for non-normal data distribution. All statistical analyses were conducted using R software (version 4.3.1; R Foundation for Statistical Computing), with a 2-tailed significance threshold of *P*<.05.

## Results

**Figure 1.**
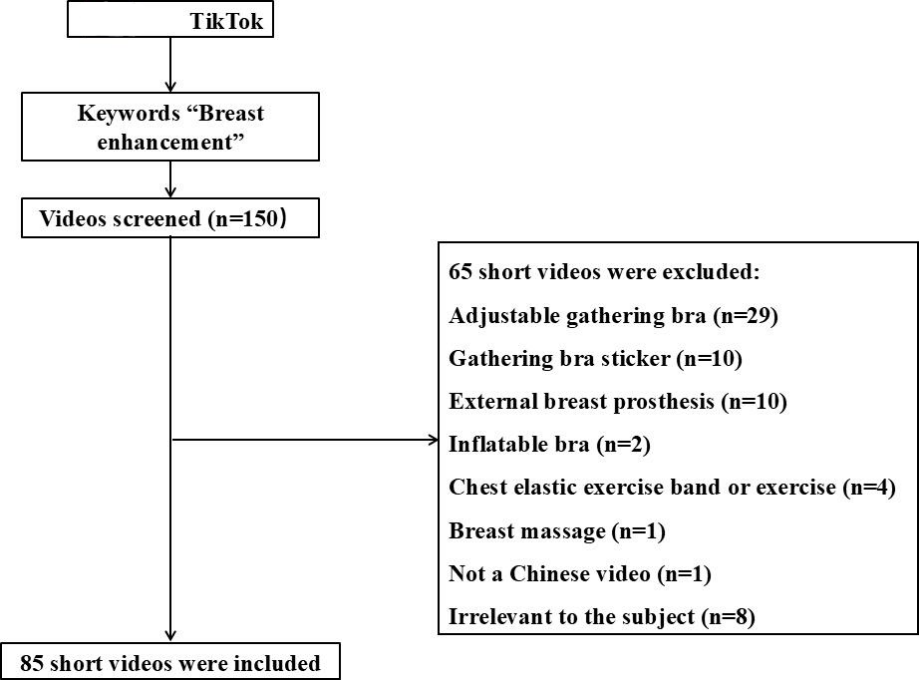
The flow chart of this study.

In this study, we included 85 TikTok videos with the hashtag breast enhancement (Figure 1), revealing patterns in uploader demographics and video content (Table 1). Most uploaders were feminine-presenting (59/85, 69.4%), followed by those who were masculine-presenting (18/85, 21.2%), and 8 uploaders (8/85, 9.4%) had ambiguous or unidentifiable gender expressions. Most of the uploaders (59/85, 69.4%) lacked professional credentials, and 16 videos (16/85, 18.8%) were created by licensed breast or plastic surgeons. This professional group comprised 12 breast surgeons and 4 plastic surgeons, with a notable gender imbalance (12 male and 4 female physicians). The credentials varied among these medical professionals: 3 attending surgeons, 9 associate surgeons, and 4 chief surgeons. Their presentation style differed from that of nonprofessionals, with 11 videos using a formal monologue format and 5 using a dialogue approach. The content focus was primarily clinical, with 12 videos specifically addressing breast augmentation procedures and 4 focusing on educational content about breast anatomy and development science. In contrast to other videos, the tone of all certified surgeons’ content was informational. Other authenticated accounts included accounts of legal professionals (3/85, 3.5%) and commercial enterprises (5/85, 5.9%).

**Table 1.** Characteristics of uploaders, product details and claims, and other video elements in videos with the breast enhancement hashtag on TikTok (N=85 videos).

Characteristics	Videos, n (%)
Influencers or featured people	
Gender expression	
Feminine	59 (69.4)
Masculine	18 (21.2)
Unidentifiable	8 (9.4)
Authentication of the uploader	
Licensed physician (plastic surgeon or breast surgeon)	16 (18.8)
Licensed lawyer	3 (3.5)
For-profit company	5 (5.9)
Licensed self-media creator	2 (2.4)
Uncertified content creator	59 (69.4)
Video style of the uploader	
Monologue with a face	41 (48.2)
Monologue without a face	1 (1.2)
Question-and-answer	12 (14.1)
PowerPoint (PPT) or educational course	2 (2.4)
Animated content	28 (32.9)
Television programs or documentaries	1 (1.2)
Featured product details and claims	
Product type (n=92 products)^a^	
Breast implant	22 (25.9)
Breast enhancement cream, essence, or essential oil	32 (37.6)
Breast enhancement patch	11 (12.9)
Breast enhancement capsule	1 (1.2)
Breast enhancement protein drink, powder, or Chinese medicinal drink	5 (5.9)
Chinese acupressure	1 (1.2)
Breast enhancement instrument	7 (8.2)
Breast enhancement needle	1 (1.2)
Homemade products	12 (14.1)
Perceived product satisfaction^b^	
Positive	71 (83.5)
Neutral	9 (10.6)
Negative	5 (5.9)
Scientific evidence of safety and efficacy	
Claims of no scientific evidence	5 (5.9)
Claims of scientific evidence	2 (2.4)
No claims of evidence	78 (91.8)
Inclusion of a “before and after” visual component	
Yes	1 (1.2)
No	84 (98.8)
Mentions of purchase methods	
Offline through the brand	0 (0)
Online through the brand directly	5 (5.9)
References a website link in their profile	5 (5.9)
Unstated	75 (88.2)
Brand name mentioned	
Yes	28 (32.9)
No	57 (67.1)

a Videos often promote more than one product in a single video.

b Videos were categorized as having a positive depiction type if they encouraged product use, a neutral depiction type if they had no stance on product use, and a negative depiction type if they discouraged product use or warned viewers of the dangers of product use.

The dominant video format was monologue-style (41/85, 48.2%), featuring the uploader’s face, which may strengthen the viewer’s connection. Other formats included animated content (28/85, 32.9%), Q&A segments (12/85, 14.1%), slideshow presentations (2/85, 2.4%), and documentary clips (1/85, 1.2%). Animated visuals appeared to be targeted toward younger audiences or those who prefer non–live-action media.

Noninvasive products were predominantly promoted, with breast enhancement creams, oils, and essences featured in 37.6% (32/76) of videos, whereas surgical options, such as breast implants, appeared in 25.9% (22/85). Other products include patches (11/85, 12.9%) and enhancement instruments (7/85, 8.2%). The focus on nonsurgical methods likely reflects either perceived lower risk or higher accessibility.

Product evaluations were predominantly positive (71/85, 83.5%), whereas critical perspectives were rare (5/85, 5.9%). However, 91.8% (78/85) of the videos lacked scientific evidence supporting product safety or efficacy. Disclaimers regarding insufficient evidence appeared in only 5.9% (5/85) of the videos. In addition, scientific validation claims were less frequent (2/85, 2.4%). Visual documentation, such as before-and-after comparisons, was nearly nonexistent, appearing in only one videos(1/85, 1.2%). Furthermore, 88.2% (75/85) of the videos lacked purchasing information, and 67.1% (57/85) omitted brand names.

Analysis of promotional strategies across the 85 TikTok videos (see Table 2) revealed distinct patterns in how breast enhancement products were promoted. More than half of the creators (50/85, 58.2%) did not claim personal use of the products they promoted. However, 41.8% of the creators shared personal experiences or surgical involvement, which may have lent credibility and authenticity to their promotional content.

**Table 2.** Promotional elements of videos with the breast enhancement hashtag on TikTok (N=85 videos).

	Videos, n (%)
Provision of testimony	
Person does not claim to have used the product	50 (58.2)
Person in the video claims to have used or surgically manipulated the product	35 (41.8)
Sponsorship	
Yes	6 (7.1)
No	3 (3.5)
Not sure, however, the screen or comment section of the video displays the product brand name	21 (24.7)
Unidentified	55 (64.7)
Tone of the video^a^	
Promotional	56 (65.9)
Informational	22 (25.9)
Confessional or opposed	7 (8.2)

a Videos were considered promotional if they marketed the product for use; informational if they contained information on the product or breast enhancement materials; confessional or opposed if they detailed their personal experiences and struggles related to breast enhancement or breast enhancement products.

Only 7.1% (6/85) of the videos explicitly disclosed sponsorship, while 24.7% (21/85) implied brand partnerships through on-screen logos or comments. The majority of the videos (55/85, 64.7%) lacked clear sponsorship markers.

Overall, the content tone of the videos was predominantly promotional, with 65.9% (56/85) advocating for the product. Information content detailing product features or enhancement methods accounted for 25.9% (22/85) of the videos, while 8.2% (7/85) presented confessional or critical perspectives, possibly aimed at audiences seeking more honest or balanced views on breast enhancement.

Analysis of engagement patterns for videos tagged with breast enhancement on TikTok (see Table 3) revealed variability in content lifespan and audience interaction. The videos averaged 36 (range 4‐418) seconds in length, adhering to the platform’s preference for brief, digestible content. The median upload time was 215 (range 5‐2264) days. However, engagement metrics showed stark contrasts. Specifically, the median likes stood at 701 (IQR 280‐2604), with extremes ranging between 38 and 133,690. The monthly like rates averaged 23.4 per 30 days, suggesting a steady yet moderate interaction.

The comment activity mirrored the variability in terms of engagement, with a median of 144 comments per video (range 0‐28,437) and 4.8 monthly comments. User saves (collections) averaged 203 per video (range 5‐22,295), translating to 6.8 monthly saves. Shares, critical for content dissemination, had a median of 350 (range 7‐213,704), with 11.7 shares per 30 days. These metrics underscore the polarized nature of engagement.

The analysis of engagement disparities between licensed physicians and uncertified content creators under the breast enhancement hashtag (see Table 4) demonstrated marked contrasts in audience interactions. The content from credentialed medical professionals achieved a median of 9761 likes (IQR 4975‐19,492). In stark contrast, nonprofessional creators garnered a median of only 701 likes (IQR 280‐2604), reflecting a comparatively limited reach.

**Table 3.** Characteristics of video reviews about the breast enhancement hashtag on TikTok (N=85 videos).

Characteristics	Median (IQR)	Min-Max
Video length	36 (24‐71)	4‐418
Duration (days)^a^	215 (112‐595)	5‐2264
Thumbs up	701 (280‐2604)	38‐133,690
Thumbs up/30 days	23.4 (9.3‐86.8)	1.3‐4456.3
Comments	144 (44‐459)	0‐28,437
Comments/30 days	4.8 (1.5‐15.3)	0‐947.9
Collections	203 (74‐826)	5‐22,295
Collections/30 days	6.8 (2.5‐27.5)	0.2‐743.2
Shares	350 (91‐1675)	7‐213,704
Shares/30 days	11.7 (3‐55.8)	0.2‐7123.5

a Duration is defined as the time (in days) since the video was posted.

**Table 4.** Comparison of video thumbs-up counts between licensed physicians and uncertified content creators uploaders on TikTok with the breast enhancement hashtag (N=85 videos).

Group	Sample, n (%)	Median (IQR)	Mann–Whitney *U*	*P* value
Uploaders			120.5	.002
Licensed physician	16 (18.8)	9761 (4975‐19,492)		
Uncertified content creator	59 (69.4)	701 (280‐2604)		

## Discussion

### Principal Findings

This study examined the dissemination of breast enhancement content on TikTok, revealing critical insights into the content of health-related messaging on short-form video platforms. The analysis demonstrated that the majority of content creators were nonprofessional, uncertified content creators, with feminine-presenting uploaders dominating the landscape. Noninvasive products, such as creams and essential oils, were promoted far more frequently than surgical options, reflecting a consumer preference for accessible and less drastic methods. Nearly all videos lacked scientific evidence to substantiate product claims, while positive portrayals of these products dominated the discourse. Notably, content from licensed physicians achieved significantly higher audience engagement compared to nonexpert creators, underscoring the influence of professional credibility in health communication. These findings highlight a platform ecosystem characterized by promotional narratives, limited transparency, and a notable absence of evidence-based guidance, raising serious concerns about the quality and reliability of breast enhancement information accessible to users.

### Comparison With Previous Work

Our research complements existing studies on breast augmentation in social media by exploring a different context and scope. Previous valuable work assessed the quality of aesthetic surgical procedure videos on TikTok using the DISCERN (an instrument for judging the quality of written consumer health information on treatment choices) scale (documenting quality scores of 1.25/5 for breast augmentation content) and investigated Instagram’s (Meta Platforms) influence on augmentation desires among young Polish women (a positive correlation with BMI and education level was identified) [35,36]. Building on this foundation, our study analyzes breast enhancement products (both surgical and nonsurgical) on Chinese TikTok. This study offers additional insights into the manner in which breast enhancement information circulates in the Chinese social media landscape, adding to our collective understanding of this topic.

### Scientific Evidence for Video Content

Scientific evidence of the safety and efficacy of breast enhancement content on TikTok appears to depend on the uploader’s expertise and how product assertions are framed. Notably, 91.8% (78/85) of the videos omitted references to scientific validation, with only 5.9% (5/85) explicitly noting evidentiary gaps. These findings mirror earlier studies demonstrating that nonprofessional creators prioritize persuasive messaging over empirical accuracy [2,37]. Our content analysis revealed 2 key gaps in the presentation of breast enhancement information. First, the videos generally did not specify particular demographic targets. The content appeared to be broadly marketed to adult women of reproductive age and married, with no videos specifically addressing teenagers, pregnant women, or postmenopausal women. This lack of audience-specific guidance is problematic given the different physiological considerations for breast tissue across life stages. The risks of misinformation are amplified by three key factors: the dominance of marketing-oriented narratives (56/85, 65.9%), the paucity of balanced dialogues addressing product safety versus potential harm, and the lack of ingredient transparency. Based on our content analysis, most videos did not explicitly disclose specific ingredients in the breast enhancement products they promoted. When ingredients were mentioned, they typically emphasized “natural” components, such as herbs or plant extracts, rather than disclosing potential hormonal content. This lack of transparency is particularly concerning since some “natural” breast enhancement products may contain phytoestrogens or other hormone-mimicking compounds without clear labeling.

These gaps collectively highlight the systemic challenges of ensuring that audiences receive transparent, evidence-based health information tailored to the specific needs of different demographic groups.

### Marketing Orientation of Video Content

As indicated by findings from the studies by Bu Bshait & Almaqhawi [38] and Pahlevan Sharif [37], the heavy emphasis on persuasive, promotional messaging—including claims that are rarely substantiated with empirical evidence—continues to dominate in this domain. This imbalance, favoring commercial interests over evidence-based education, limits audiences’ ability to make informed decisions about these products or procedures. Furthermore, the lack of honest or critical communication of risks and realistic outcomes exacerbates the challenge of navigating the potential harms associated with nontransparent and scientifically unsupported claims. Beyond commercial focus, social media also perpetuates shallow aesthetics.

### Beauty Visions and Health Literacy in Social Media

Similarly, this study’s results resonate with the conclusions of Peltzer et al [14], who found that social media often perpetuates shallow beauty ideals, largely avoiding critical discussions or complex dialogue around the implications of promoting such standards. The content’s tendency to simplify or glorify beauty interventions remains evident across platforms, including TikTok. Despite the presence of licensed physicians contributing to the health-related discourse on TikTok, more balanced and nuanced discussions covering the potential complications, postoperative realities, and long-term efficacy of breast enhancement products or procedures remain sparse. Although health care professionals tend to garner higher engagement metrics in social media spaces, consistent with findings in digital health communication research highlighting the credibility associated with expert voices [39,40], this does not always translate into greater comprehensiveness or depth in the information provided. In addition, in the future, we could establish straightforward, publicly available, standardized frameworks for evaluating breast satisfaction that could improve health literacy, such as developing verified metrics and guidelines for assessing breast health and aesthetics and helping viewers differentiate between scientifically grounded recommendations and purely promotional content. Using evidence-based instruments could improve societal comprehension of aesthetic health, foster informed decision-making, and reduce dependence on non-academic media, such as films, music videos, or literature, for guidance. Such initiatives could guide regulatory strategies and platform policies to combat misinformation hazards while advancing equitable public health outcomes.

### Audience Engagement in Video Content

Audience interaction patterns differed markedly between content from medical professionals and non-expert creators. Videos by licensed physicians attracted a median of 9761 likes, substantially exceeding the 701 likes for uncertified content creators. This suggests that professional credentials enhance perceived reliability and drive heightened audience engagement. This aligns with previous work demonstrating that institutional affiliations and specialized knowledge bolster credibility in digital health communication [39]. However, engagement measures, including likes, comments, and shares, showed considerable variability across all uploaders, indicating that viewer responses are shaped by elements such as production quality, topical relevance, and creator expertise.

### Theoretical and Practical Implications

Breast enhancement content is widely followed on short video platforms, paralleling rising societal interest in aesthetic health and self-perception. In the digital era, users increasingly turn to social media apps like TikTok to gather medical and aesthetic information, share personal experiences, and seek advice. Our study reveals that content from licensed physicians on TikTok attract higher engagement, suggesting viewers value professional insights. However, challenges persist, including a lack of scientific evidence, reliance on promotional content, and anecdotal claims, particularly when algorithms favor catchy clips over in-depth content.

### Theoretical Perspectives

Our analysis draws upon the following theoretical perspectives to understand potential viewer impacts: the content analysis of breast enhancement videos reveals a predominant focus on standardized aesthetic criteria centered primarily on breast size. Through the lens of “objectification theory” [41], this content orientation may foster viewers’ self-objectification, a psychological process in which individuals internalize an observer’s perspective and evaluate their bodies as objects to be assessed by others. This may shift viewers toward valuing breasts predominantly for their aesthetic appearance rather than recognizing their biological and functional significance (such as breastfeeding).

In addition, from the perspective of “cultivation theory” [15], consistent and repeated exposure to such idealized breast enhancement content across the platform may gradually alter viewers’ perception of normal breast morphology, potentially cultivating unrealistic expectations about what constitutes typical or desirable breast appearance. This theoretical framework helps explain how social media platforms can shape users’ understanding of the body through systematic exposure to particular aesthetic standards. Studies further suggest that breast enhancement advertising content often promotes a “quick fix” mindset [2,42,43], which, when algorithmically amplified, normalizes rapid but potentially harmful body modifications. By perpetuating idealized images and commercial messaging, TikTok’s recommendation engine may reinforce body dissatisfaction and foster normative beliefs that breast implants and breast enhancement creams, essences, or oils are the primary route to self-improvement.

### Practical Implications

These theoretical insights inform our practical recommendations for mitigating potential negative impacts. Therefore, we propose a dual-mode content review and certification of creators in both AI and manual modes, along with transparent referencing of expert sources. Beyond this proposal, TikTok has implemented certain measures to address body image issues, including oversight of images on the platform, censorship of advertisements for illegal products, and removal of harmful content (such as content supporting eating disorders, appearance-based bullying content), and age restrictions on weight loss advertisements [34,44-48]. Nevertheless, platforms could improve oversight of sensitive health information, implement clearer sponsorship disclosures, and ensure that products promoted are reliable.

Additional safeguards, including mandatory health disclaimers and age verification for high-risk hashtags, could further protect younger audiences. Algorithmic adjustments to limit the virality of unverified claims, combined with evidence-based educational material, may bolster user health literacy and encourage more informed decisions about breast enhancement options.

By integrating objectification and cultivation theories with the “quick fix” concept, this study highlights how social media–driven breast enhancement trends can shape body image perceptions. Corresponding practical steps—such as stricter content regulation, transparent labeling, and enhanced user protections—seek to alleviate potential harms and foster a healthier, more informed internet-based community.

### Limitations

This study has some limitations. First, the analysis was limited to TikTok, a single platform, and focused on Chinese-language content from the Chinese version of TikTok, leading to platform, geographic, and language constraints and restricting its generalizability to other platforms, regions, or linguistic contexts. Second, the dynamic and fast-paced nature of short video platforms means that content evolves rapidly, potentially affecting the relevance of the findings over time. Third, the manual classification of video characteristics, such as tone and product satisfaction, introduces researcher bias and reduces the objectivity of the analysis. Fourth, our study analyzed the content characteristics and engagement patterns but did not establish evidence of an association between sponsored breast enhancement content and viewers’ actual willingness to pursue such products or procedures. Finally, the study is limited by ethical and cultural sensitivities, as its focus on Chinese language content may reflect specific cultural attitudes toward beauty standards and cosmetic surgery. As such, these findings may not apply to other cultural contexts where societal norms and perceptions of breast enhancement differ.

Future research could refine the following areas: (1) Multi-platform analysis: expanding the scope to include other short-video platforms, such as Instagram Reels, YouTube Shorts, and Bilibili, would enable comparative analyses of how breast enhancement content is presented and engaged with across different digital ecosystems; (2) Artificial intelligence–driven content analysis: leveraging machine learning and natural language processing tools could automate the classification of video characteristics, including tone, sentiment, and themes, thereby reducing subjective bias in content analysis; (3) Longitudinal studies tracking changes in breast enhancement content and engagement patterns over time could account for the dynamic nature of social media trends and platform algorithms. In addition, cross-sectional studies incorporating more labels could provide a broader scope of analysis, especially by including videos that overlap under different tags; (4) Cross-cultural comparisons: investigating how breast enhancement content varies across geographic regions, linguistic contexts, and cultural backgrounds would be particularly valuable to understand the differences in beauty standards, promotional strategies, and consumer responses; such comparisons could reveal important insights into how cultural factors influence perceptions and representations of breast enhancement on social media platforms globally; (5) Further research should examine both the psychological impact of breast enhancement content on viewers’ body perception and mental well-being (particularly among younger audiences) and the ethical considerations surrounding content creator responsibility, disclosure standards, and platform governance in health-related social media environments; and (6) Influencer and brand analysis: future studies should investigate the role of influencers, medical professionals, and breast enhancement brands in shaping breast enhancement narratives on TikTok, including analyses of sponsored content, promotional strategies, and partnership disclosures. Specifically, research using structured internet-based surveys or experimental designs could evaluate the direct association between users’ exposure to sponsored breast enhancement content and their willingness to purchase such products or undergo procedures.

### Conclusions

Our findings highlight TikTok’s dual role as both a democratizing force and a potential source of misinformation in the public perception of breast enhancement. The platform increases accessibility to health-related content; however, its dominance by nonprofessional creators risks the spread of idealized beauty standards and unverified claims. Furthermore, the study’s findings emphasize the need for licensed medical professionals to actively create evidence-based content to counter misinformation and foster balanced discussions about risks and benefits. Platforms must collaborate with health care providers and regulators to implement verification systems, flag misleading content, and promote transparency, such as through sponsorship disclosures. In addition, prioritizing digital literacy and ethical guidelines can help mitigate harm to vulnerable audiences, such as adolescents influenced by unrealistic portrayals. The integration of medical expertise into viral health communication on TikTok and similar platforms can transform these platforms into trustworthy educational tools that empower users to make informed decisions based on scientific integrity. Bridging this gap is crucial for aligning the influence of social media with public health priorities.

## Abbreviations

Q&A: question and answer
